# Micro-nano robots for treatment of eye diseases

**DOI:** 10.3389/fchem.2025.1553461

**Published:** 2025-04-10

**Authors:** Haocheng Wang, Jingxue Sun, Xiaona Zhang, Yingjie Zhou, Yaguang Zhang, Yanan Ni, Minnan Wang, Yanmeizhi Wu, Lulu Sun, Xiaoyu Zhao, Hong Qiao

**Affiliations:** ^1^State Key Laboratory of Robotics and System, Harbin Institute of Technology, Harbin, China; ^2^ Suzhou Research Institute of HIT, Suzhou, China; ^3^ Department of Endocrinology, The Second Affiliated Hospital of Harbin Medical University, Harbin, China; ^4^ Department of Anesthesiology, the Second Affiliated Hospital of Harbin Medical University, Harbin, China

**Keywords:** micro-nano robots, eye diseases, drug delivery, on-target drug delivery, applications

## Abstract

In recent years, micro-nano robots have found extensive applications in the field of medicine. Among these, micro-nano robots demonstrate significant potential for the treatment of eye diseases. Micro-nano robots can penetrate eye tissue barriers to directly target the posterior segment of the eye for precise drug delivery, enabling non-invasive or minimally invasive treatment. This review explores the primary fabrication methods and propulsion mechanisms of various micro-nano robots, and their applications in treating various eye conditions, offering inspiration and guidance for advancing the use of micro-nanorobots in ophthalmic therapies.

## 1 Introduction

Micro-nano robot is a kind of robot with a scale between microns and nanometers, which can complete a series of activities such as execution, perception and decision-making at the micro-nano scale, and has been widely used in the medical field in recent years. Since the father of micro-nano robotics Toshio Fukuda created the world’s first nanorobot at the beginning of the twenty-first century (Fukuda et al., 2004), micro-nano robots have been widely used in the field of biomedicine, more and more technologies and means can be applied to micro-nano robot technology, the rapid development of micro-nano robot research has also driven the continuous progress of other related disciplines.

Different from macroscopic robots, micro-nano robots have the advantages of high precision, high flexibility and wide adaptability, and the application of micro-nano robots to carry out targeted drug treatment and non-invasive surgery have shown important application prospects in the field of life and health (Zhang et al., 2024). In ophthalmic treatment, due to the complex structure of the eye and the high risk of surgery, there are extremely high requirements for the accuracy of the doctor’s operation. The advantages of micro-nano robots such as refined operation degree and precise treatment show their great potential in ophthalmic treatment (Barbot et al., 2019). Due to the presence of multiple barriers in the eye, traditional drug delivery methods are unable to effectively transport medications to the posterior segment of the eye and may potentially cause side effects (Baumal et al., 2020). With the advancement of nanotechnology, micro/nanorobots capable of enhancing drug permeability, stability, and targeting specificity offer a new direction for ocular drug delivery.

## 2 Manufacturing and design of micro-nano robots

Researchers have applied insights from the unique movement patterns of organisms in nature to artificial micro-nano structures, controlling them to execute precise movements in low Reynolds number liquid environments for purposes such as non-invasive surgery, targeted drug delivery, cell manipulation and separation, and biological imaging. These micro-nano structures are referred to as micro-nano robots (Xu et al., 2016). The manufacture of micro-nano robots involves multiple disciplines, including materials science, mechanical engineering, biology, etc. In ophthalmic treatment, micro-nano robots need to be flexible and stable enough to be able to perform precise operations inside or on the surface of the eye. In addition, micro-nano robots also need to have good biocompatibility to reduce damage to eye tissue.

### 2.1 Manufacturing process of micro-nano robots

**FIGURE 1:**
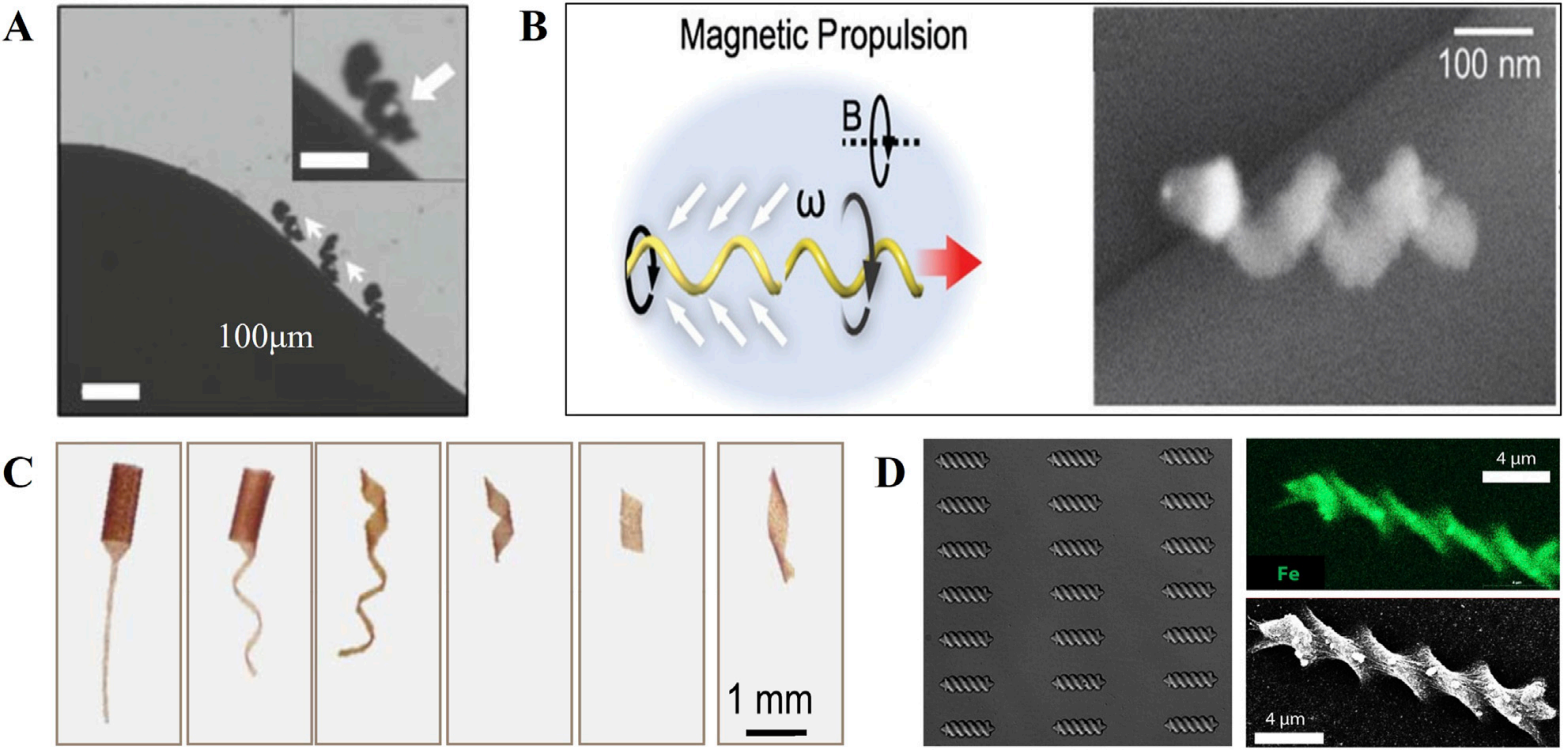
Micro-nano robots with micro-helix structure. **(A)** Biodegradable soft helical microswimmers (Dong et al., 2020). Reproduced with permission, Copyright 2020, WILEY-VCH Verlag GmbH & Co. KGaA, Weinheim. **(B)** Magnetic helical nanorobots (Wu et al., 2020). Reproduced with permission, Copyright 2020, Royal Society of Chemistry, Copyright 2014, American Chemical Society. **(C)** Bioinspiredmicroswimmers inspired by bacteria (Huang et al., 2019). Reproduced with permission, Copyright 2019, he American Association for the Advancement of Science. **(D)** Biodegradable hydrogel microrobotic swimmers (Ceylan et al., 2019). Reproduced with permission, Copyright 2019, American Chemical Society.

Most of the design inspiration of micro-nano robots comes from organisms in nature, and the mechanical device is used to simulate the morphological characteristics, structural characteristics, functional properties, etc. Of microorganisms that already exist in nature. As Figure 1A, common micro-nano robots are spiral, linear, tubular, Janus granular, spherical, gear-shaped, capsule-shaped (Dong et al., 2020) (Peng et al., 2020). At present, the widely used synthetic micro-nano robot methods are: chemical synthesis method (Hoop et al., 2018), deposition method (Wang, 2013), self-assembly method (Wu et al., 2015), curling method (Yin et al., 2012), template method (Lyu et al., 2021), 3D printing technology (Wang et al., 2018; Zhang et al., 2019).

Brownian motion and the Reynolds number are two crucial factors that govern the motion of micro-nano robots in fluid environments. The Reynolds coefficient is inversely proportional to viscosity and proportional to inertia, so the Reynolds coefficient has become a necessary consideration for the resistance of micro-nano robots when developing. With the reduction of the size of micro-nano robots, Brownian motion becomes another determining factor, which affects the direction of motion of micro-nano robots. Studies have shown that the microhelix structure is an ideal motion model in a low Reynolds number environment, as Figures 1B–D, so most micro-nano robots are designed as micro-helix (Wu et al., 2020; Huang et al., 2019; Ceylan et al., 2019). In the rotating magnetic field of electromagnetic propulsion, The micro-nano robot also generates driving force in the forward direction with rotation it is considered that the motion of the microhelix is affected by the combination of viscous force and moment balance, and the fast movement speed is at high magnetic frequencies. Therefore, this microhelix structure can reduce the drag coefficient and achieve the fastest motion speed in a low Reynolds number environment (Wang et al., 2018). At the same time, this microhelix structure also has the potential to be applied in other fields (Mourran et al., 2016).

There are various synthesis methods for micro-nano robots. The principle is as follows, chemical synthesis: This is a relatively simple preparation method, by changing the conditions and synthesizing robots with the help of chemical reactions. Deposition method: In the field of micro-nano robots, the vapor deposition method and electrodeposition method in the deposition method are mainly used, and the principle is that the electric or vapor phase deposits materials with specific functions on the template. Micro-nanorobots prepared using this method exhibit diverse morphologies and low production costs. Self-assembly method: based on the electrostatic interaction, the polymer with positive and negative charged particles interact with each other to spontaneously form an ordered and regular structure. Micro-nanorobots prepared using this method possess self-repair capabilities. Curling method: material is introduced on the substrate deposited sacrificial layer, etching of the lower layer, the sacrificial layer is etched so that the upper material is bent into a tube. Micro-nanorobots prepared using this method exhibit high controllability and reproducibility. Template method: Using organisms or objects with good shape and performance as templates, the template is removed by external stimulation or sintering. Micro-nanorobots based on biological templates offer the potential for high biocompatibility. 3D printing technology: A technology that uses a printer to print objects layer by layer using a specific material using collected data. Since laser 3D printing technology can create various 3D micro-nanostructures solely through clean photopolymerization effects, it holds broad prospects for precise, high-resolution, and large-scale manufacturing of micro-nanorobots.

### 2.2 Design of micro-nano robot for ophthalmic treatment

**FIGURE 2:**
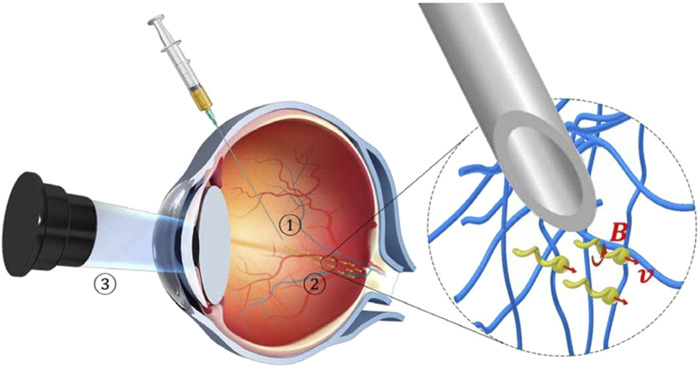
Schematic of the three-step targeted delivery procedure used for the slippery micropropellers (Wu et al., 2018). Reproduced with permission, Copyright 2018, The American Association for the Advancement of Science. ① Injection of the micropropellers into the vitreous humor of the eye. ② Magnetically driven long-range propulsion of the micropropellers in the vitreous toward the retina. ③ Observation of the micropropellers at the target region near the surface of the retina by OCT.

Although the most commonly used administration method for the treatment of eye diseases is topical administration, the physiological obstacles of local absorption are large, the absorption rate of drugs is low, only about 1%–10% of topical drugs can penetrate the eyes, so it is necessary to administer high-frequency and increase the dose (Ullrich et al., 2013), and micro-nano robots that can enter the ocular administration have become the new hope to give local administration of the eye. Some studies have shown that micro-nano structures can effectively pass through the vitreous (Wu et al., 2018; Liu et al., 2024). The first micro-nano robot (Figure 2) to penetrate the vitreous to reach the retina used a perfluorocarbon surface coating that reduce the interaction of the propeller with biopolymers such as collagen bundles present in the vitreous. They demonstrated that propulsion in biological media should meet two main criteria: ① particle size and shape could be effectively passed through the macromolecular network; ② Reduce the interaction between propellers and biopolymer networks.

**FIGURE 3:**
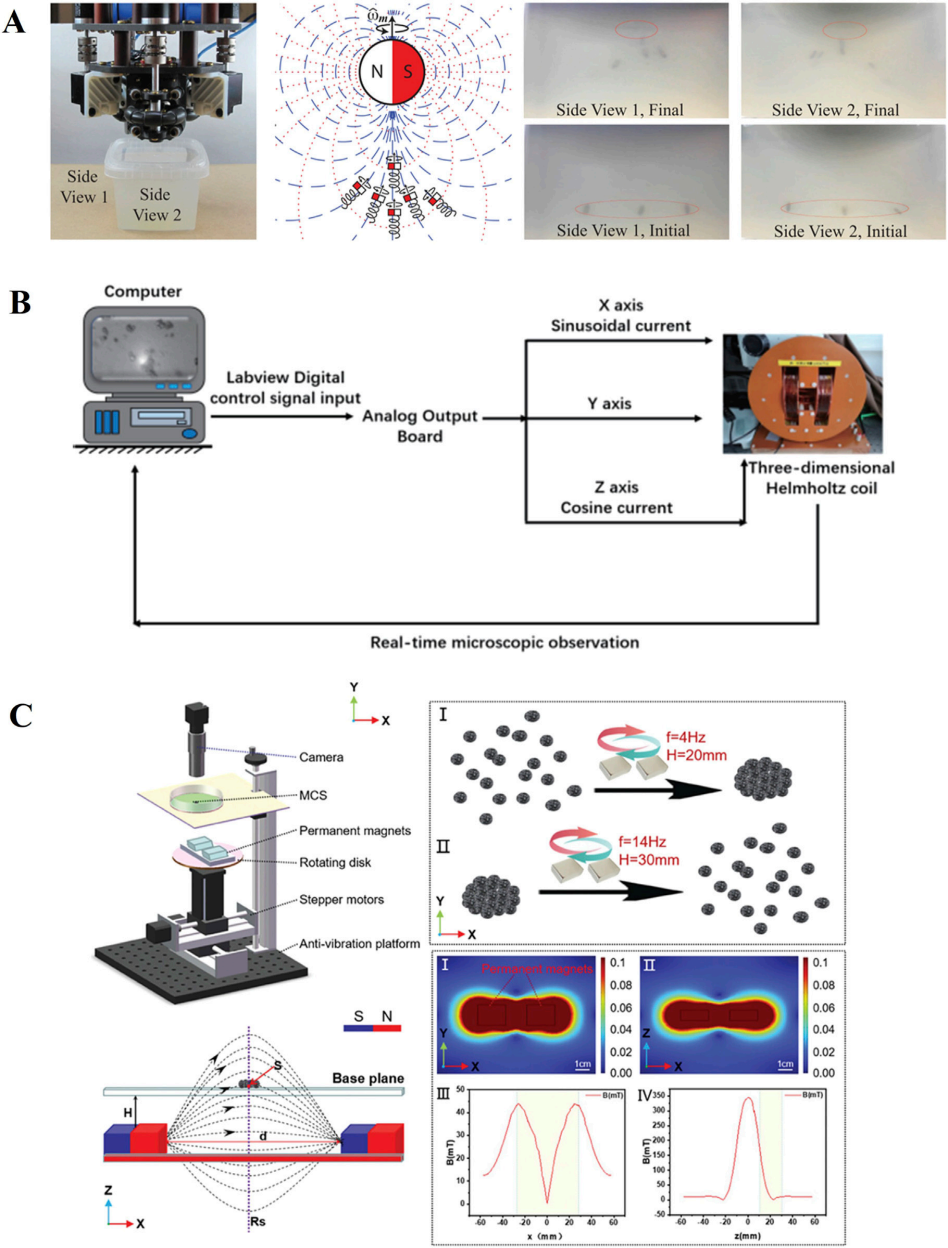
Magnetically driven micro motor: **(A)** exogenous magnetic field drive (Chaluvadi et al., 2020). Reproduced with permission, Copyright 2020, IEEE. **(B)** Rotating magnetic field drive (Feng et al., 2019). Reproduced with permission, Copyright 2019, IEEE. **(C)** Magnetic tweezers (Su et al., 2024). Reproduced with permission, Copyright 2024, Wiley–VCH GmbH.

Magnetic drive is currently the most widely used method (Figure 3), it has the advantages of effectively penetrating biological tissues and remote drive, and there are successful cases using exogenous magnetic field drive (Chaluvadi et al., 2020), rotating magnetic field drive (Feng et al., 2019) and magnetic tweezers (Su et al., 2024) driving methods. By adjusting the intensity and frequency of the magnetic field, it is possible to manipulate micro-nanorobots to assemble, navigate, and overcome obstacles in complex environments (Zhang et al., 2023; Li et al., 2023; Yu et al., 2022; Ji et al., 2021; Zhang et al., 2025). The optical drive has the advantages of remote control, low noise and spatiotemporal selection ability, and the eye itself is a light-transmitting tissue, which has an innate advantage in avoiding the damage caused by the heat generated by the light to the tissue (Chen et al., 2022). In the structure of the eye, the aqueous humor circulation system and the vitreous body can both be considered as low Reynolds number environments. Among them, the vitreous body has a higher viscosity, making it more difficult for micro-nanorobots to penetrate. Sonic propulsion can be driven in such a way that greater propulsion can overcome the low Reynolds number state dominated by viscosity, and low-frequency ultrasound is less harmful to biological samples (Yang et al., 2018). In addition, the effects of ultrasound on micro-nanoparticles are also widely applied in environmental management, metallurgy, and other industries (Yu et al., 2020; Yu et al., 2024).

In terms of energy supply, corrosive chemical fuels is one of the typical energy supply methods (Yang et al., 2018), and some teams have designed micro-nano robots that use their own glucose as energy supply (Kutorglo et al., 2021). The introduction of biomaterials make bio-driven method very biocompatible (Zheng et al., 2023), but due to the presence of the blood-eye barrier, bacteria-based micro-nanorobots may not be able to enter the eye. The hybrid drive method can eliminate the above shortcomings, so the driving method of the eye micro-nano robot can use hybrid drive.

**FIGURE 4:**
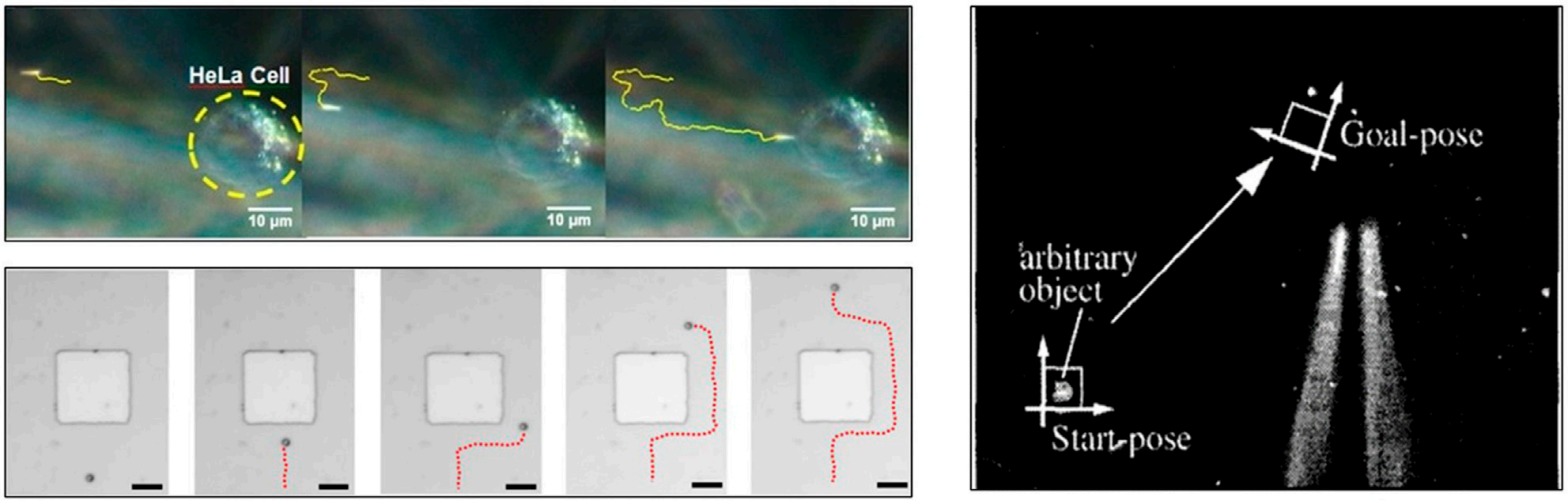
Feedback of micro-nano robots is based on visual feedback systems (Ahmed et al., 2013; Li et al., 2017; Pappas and Codourey, 1996). Reproduced with permission, Copyright 2013, American Chemical Society, Copyright 2017, American Chemical Society, Copyright 1996, IEEE.

At present, most of the real-time feedback of micro-nano robots is based on visual feedback systems (Figure 4) (Ahmed et al., 2013; Li et al., 2017; Pappas and Codourey, 1996) and force feedback (Kim et al., 2008), and for organs with fine structures such as eyes, precise positioning is more important. How to make micro-nano robots make flexible and accurate movements and intelligent three-dimensional control of their movements is still a problem to be solved (Yang et al., 2018).

### 2.3 Challenges faced by micro-nano robots in the treatment of eye diseases

Although micro-nano robots are small in size and easy to pass through the organizational structure, they can integrate signal perception, collection and processing (Yang et al., 2018) but their functional diversity and signal feedback system still have shortcomings. As a medical tool, it is necessary to find suitable materials for micro-nano robots that are both tissue-compatible and highly safe, so that they are neither collectively rejected nor adversely affected by the organism. In addition, micro-nano robots should also pass through the biological barriers present in the eyes, so that they can deliver drugs to the corresponding target sites and perform therapeutic operations on the targets. As a high viscosity environment of intraocular vitreous the micro-nano robot completed by the task should also have the ability of automatic degradation or be equipped with a recycling structure, so that it will not cause adverse effects or damage to the body after completing the task (Yang et al., 2018). Animal experiments of retinal targeted delivery have not yet entered clinical trials on a large scale, and there is a lack of data on the long-term efficacy and safety of micro nano robots in the treatment of eye diseases (Wu et al., 2018). The complex internal environment in the human body will also have an impact on the movement and operation of micro-nano robots, such as difficulty in movement, operation obstacles, and loss of direction.

Micro-nano robots require strong technical support, which requires new energy conversion mechanisms, more powerful wireless drive and control methods, and more reasonable manufacturing technologies (Yang et al., 2018). There are still several critical issues that need to be resolved before micro-nano robots can be used clinically in the treatment of eye diseases. Animal experiments targeting retinal delivery have not yet progressed to large-scale clinical trials, and there is a lack of data on the long-term efficacy and safety of micro- and nanorobots in the treatment of ocular diseases. The manufacturing of micro-nano robots requires more commercial enterprises to participate in jointly completing the transition of micro-nano robots from theory to practice, and solving the problems of technology, materials and costs (Yang et al., 2018).

However, as a microstructure that can be finely operated, it has high clinical application value and broad future development prospects, and the above problems can be solved with the development and further development of research.

## 3 Application of micro-nano robots in the treatment of eye diseases

Micro-nano robots have a variety of applications in the treatment of eye diseases. In the treatment of ocular tumors, micro-nano robots can accurately locate tumors, perform minimally invasive surgery, and reduce damage to surrounding healthy tissues. In the treatment of ocular inflammation, micro-nanorobots can release anti-inflammatory drugs to effectively control inflammation. In eye injury repair, micro-nano robots can perform fine sutures to promote wound healing. In addition, micro-nano robots can also be used for the diagnosis of eye diseases and improve the accuracy of diagnosis.

### 3.1 Application of micro-nano robots in the treatment of ocular tumors

Eye cancer includes malignant tumors of the eyelids, conjunctiva, various tissues of the eyeball, and ocular adnexa, such as retinoblastoma, choroidal melanoma, and basal cell carcinoma of the eyelid, posing significant harm. Due to the uniqueness of the eye structure, traditional surgical removal is challenging to achieve complete cure and can cause severe physical and psychological harm to patients. Micro-nanorobots offer a new therapeutic approach for the targeted drug delivery to tumor tissues in the treatment of ocular cancer. Some scholars proposed biomedical micro-nano robot example to use monocytes (such as macrophages) as microdrivers and microsensors for solid tumor active drug delivery systems (Dai et al., 2021). Monocytes have a sensory receptor that recognizes foreign bodies and inflammation in the living body, and are self-actuating, allowing it to move from the bloodstream to the corresponding target site in the tissue when it detects a signal of infection or inflammatory response. And monocytes can phagocytose foreign bodies and differentiate into macrophages or dendritic cells, and microrobots with monocytes as the carrier will show powerful effects in tumor treatment (Li et al., 2003; Liu et al., 2004).

**FIGURE 5:**
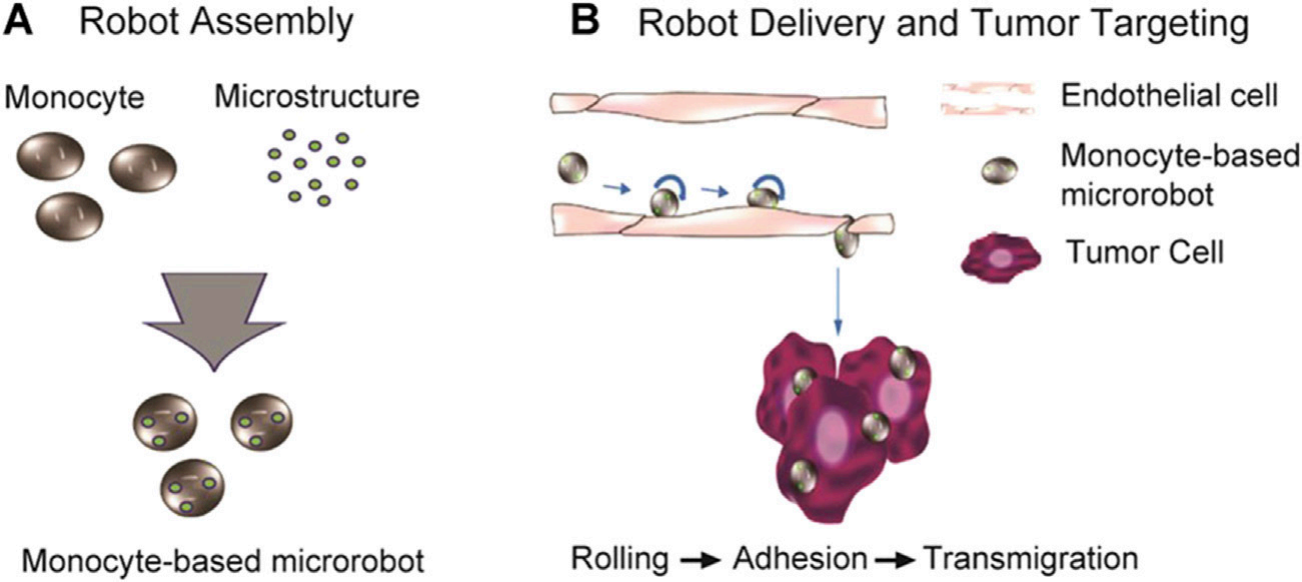
Monocyte-based microrobot with tumor targeting property (Park et al., 2014). Reproduced with permission, Copyright 2014, Wiley Periodicals, Inc. **(A)** Schematic representation of the fabrication of monocyte-based microrobot and **(B)** tumor targeting concept of monocyte-based microrobot transmigrating over endothelial cells.

Monocytes-based microrobots can show migration properties to tumor cell lysates and tumor-containing tissues, such as MCP-1, MCF7 cell lysates and MCF7 containing alginate spheroids (Park et al., 2014). Therefore, if the phagocytosed droplets are replaced with therapeutic drugs that target tumors, monocytes-based microrobots can be used as important tools for biomedical therapy. In addition, in order to overcome the rejection of foreign monocytes by the immune system, monocytes isolated from individual living animals should be used to develop a microrobot based on homogeneous monocytes (Figure 5) (Park et al., 2014).

### 3.2 Application of micro-nano robots in the treatment of ocular sterilization

**FIGURE 6:**
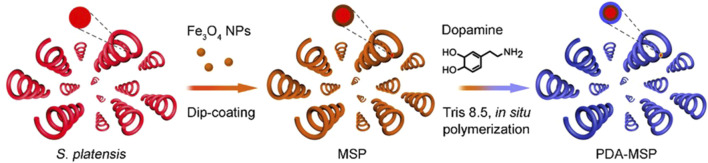
Schematic of the fabricating process based on the Spirulina platensis, including the Fe_3_O_4_ NP dip-coating and *in situ* DA polymerization (Xie et al., 2020). Reproduced with permission, Copyright 2020, American Chemical Society.

There are complex inflammatory mechanisms involved in the pathogenesis of eye diseases. Due to the complex ocular biological barrier, targeted bactericidal therapy has become a more meaningful treatment approach. A team has developed and tested a multifunctional magnetic microswimmer that introduces a polydopamine (PDA) coating (such as magnetized Spirulina 72 h (MSP-72)h, dipping MSP for 72 h) on spirulina micro-nano robots (Figure 6) (Xie et al., 2020). The coating process uses a simple but versatile DA self-polymerization process with the advantages of low cost and scalability. The microswimmers manufactured (such as PDA-3h) successfully integrate many core functions and are expected to solve the problems existing in medical micro-nanorobots.

The study used photothermal antimicrobial therapy to observe antimicrobial outcomes. After the detection of multi-drug resistant *Klebsiella* Pneumonie (MDR KP), the photothermal effect enhanced by PDA is used to kill it, and the killing or antibacterial effect at different concentrations is evaluated by its corresponding bacterial viability. Lower values indicate higher efficacy and *vice versa*. With the increase of PDA-3h concentration, its survival rate gradually decreased to less than 1% at 400 μg/mL. In contrast, in the absence of NIR light irradiation, the corresponding activity value of all concentrations of bacteria was above 90%, which means that the antibacterial effect of PDA-3h itself is almost negligible. For effective photothermal antimicrobial, the target favorable temperature should be above 50°C (He et al., 2019; Ray et al., 2012). The temperature of the 400 μg/mL sample did not reach 65°C until 4 min after irradiation, which has a very high antimicrobial effect and kills almost all MDR KP. Such high temperatures may inhibit bacterial metabolism and cause the death of MDR KP through denaturing enzymes and destruction of membrane proteins/lipids, but there are still great obstacles to the application of such high temperatures to ophthalmic treatment.

In live/dead staining, green fluorescent SYTO nine and red fluorescent propidium iodide (PI) dyes are used to label “live” and “dead” MDR KP, respectively. PI dyes play a role in determining membrane integrity by inserting nucleic acid sequences within cells (Ray et al., 2012). Once the cells are stained red by the PI dye, this indicates some damage on the cell membrane. Thus, in Confocal Laser Scanning Microscopy (CLSM) images, the presence of erythrocytes validates the mechanism of membrane damage associated with MDR KP. Scanning electron microscopy (SEM) images have also shown that the surface of the MDR KP, which should be intact and smooth, is wrinkled, collapsed, and even lysed.

### 3.3 Application of micro-nano robots in the treatment of eye injuries

The treatment of eye injuries requires the use of micro-nanorobots with high targeting capability and biocompatibility. Some researchers have designed a kind of nanoparticles, which leveraged poly (l-histidine) surface and surface roughened ceria nanocages (SRCNs) to address chemical corneal injuries effectively (Yang et al., 2023). The SRCNs’ controlled roughness improved cellular uptake and therapeutic efficiency while maintaining optimal ocular biocompatibility. The poly (l-histidine) surface enhanced corneal penetration and allows targeted drug release in the acidic environment caused by tissue damage. SRCNs are loaded with acetylcholine chloride (ACh) and SB431542, which synergistically promote wound healing, inhibit fibrosis, and suppress inflammation. In addition, hydrogel particles are often used for drug delivery to facilitate tissue repair (Li et al., 2025). Those provides a new material for micro-nano robot to treat eye injury.

## 4 Conclusion

With their small size, micro-nano robots have the potential to overcome the physiological barriers within the eye, such as the blood-aqueous barrier and the blood-retinal barrier. Compared with traditional treatment methods, micro-nano robots have made more advances in the treatment of eye diseases. Non-invasive local drug administration is the best choice for many eye diseases. Although it is a challenging task to safely and effectively deliver drugs to the diseased areas of the eye, researchers have been continuously designing new micro-nano robots with the hope of transitioning from laboratory to clinical use. In the future, safe and effective nano-robots will revolutionize the treatment of eye diseases.

Despite the advantages of micro-nanorobots in the treatment of eye diseases, there are still challenges and issues that need to be addressed. As a medical device, the safety of micro-nano robots should also be used as the main influencing factor to affect the development of micro-nano robots, such as tissue compatibility problems, rejection problems, waste recycling problems, etc. Considering the structural differences between the human eye and animal eyes, conducting clinical trials poses a significant challenge for the application of micro-nanorobots in treating ocular diseases. Additionally, the manufacturing process for micro-nanorobots is costly, and the yield rate remains limited. Achieving scalable mass production of micro-nanorobots will be a problem that needs to be resolved in the future. Despite these challenges, with the advancement of technology, the application of micro-nano robots in ophthalmic treatment will be more extensive and more accurate, bringing better treatment results to patients.

## Abbreviations

MCP-1, Monocyte chemotactic protein-1; MCF7, Michigan Cancer Foundation7; PDA, Polydopamine; MSP, Magnetized Spirulina; DA, Dopamine; MDR KP, Multi-drug-resistant *Klebsiella pneumoniae*; PI, Propidium iodide; CLSM, Confocal Laser Scanning Microscopy; SEM, Scanning electron microscopy; SRCNsSurface roughness-controlled ceria nanocages; ACh, acetylcholine chloride.
